# Supplementary material for: Are carbon emissions trading and green financial instruments synergistic? -Comprehensive quantitative research based on content analysis
Source: PLoS One. 2024 Mar 7;19(3):e0298601. doi: 10.1371/journal.pone.0298601 (PMC10919601; doi:10.1371/journal.pone.0298601)
Supplement: S1 Data — (ZIP) [file pone.0298601.s001.zip › Data/地级市Z_F工作报告.zip!/╜¡╦╒/╠⌐╓▌╩╨/└┤╫╘╣1⁄2╓┌║┼í╛┬φ┐╦╩2╛▌═°í┐_2004.docx]

各位代表：

现在，我代表市人民政府，向大会作工作报告，请予审议，并请市政协委员和其他列席人员提出意见。

一、2003年工作回顾

过去一年，我们在中共泰州市委领导下，以邓小平理论和“三个代表”重要思想为指导，紧紧围绕力争“五年总量翻番、八年全面小康”的奋斗目标，解放思想，凝心聚力，扎实苦干，努力消除“非典”疫情和洪涝灾害带来的不利影响，全面完成了市二届人大一次会议确定的经济和社会发展的各项目标任务。

经济增长速度加快，人民生活进一步改善。预计实现国内生产总值580亿元，人均国内生产总值11492元，分别增长13.4%和13.2%，增速均为建市以来最高水平。其中，第一产业增加值73亿元，增长4%；第二产业增加值299.7亿元，增长17.4%；服务业增加值207.3亿元，增长11.7%。农业结构调整取得成效，优质、高效农产品增加，林业面积新增14.5万亩。扶持农业产业化经营龙头企业力度加大。农业利用“三资”超过9亿元。市农业开发区启动建设，并被列为省级现代农业综合开发示范区。农业标准化工作继续推进，农产品质量体系建设得到加强。农村劳动力转移加快，新增劳务输出10万人。去年淮河流域发生特大洪涝灾害。我市里下河地区广大干部群众顽强拼搏，团结防汛，取得了抗洪斗争的全面胜利。工业经济提速增效。预计列统工业企业销售收入640亿元、利税66亿元、利润32.5亿元，同比分别增长29%、37.3%和52.4%。重点骨干企业支撑作用增强，30强企业预计实现销售收入265.7亿元、利税40亿元、利润21.5亿元，分别增长38%、68.5%和70%。工业技改投资首次超过100亿元，比上年净增33亿元。年初确定的120个重点技改项目全部开工建设，其中80个项目已经竣工投产。乡镇工业园区建设步伐加快，预计完成销售收入174亿元，增长50.9%。10个乡镇工业园区被评为国家级、省级工业小区或科技园区。建筑业持续发展。预计完成建筑业总产值241.3亿元，增长15%。建筑规模企业进一步壮大，产值超过5亿元的企业达到6家。服务业平稳发展。房地产业、餐饮业快速增长，中介服务、社区服务业形势喜人。预计全社会消费品零售总额172.6亿元，增长11.7%。财税金融工作取得新成绩。完成财政收入66.3亿元，增长24.7%。年末本外币各项存款余额608亿元，各项贷款余额353.3亿元，比年初分别增长21.1%和21.6%。人民生活水平稳步提高。预计城镇居民人均可支配收入8500元，农民人均纯收入4065元，分别增长9.1%和6%。市区人均住房使用面积19平方米，全市农民人均住房使用面积41.4平方米，分别比上年增加1.8平方米和0.8平方米。就业再就业和社会保障工作进一步加强，全市城镇新增就业人员2万人，下岗失业人员再就业1.6万人，“两个确保”和城镇居民最低生活保障制度得到落实，企业退休人员在全省率先全面纳入社会化管理轨道。农村自来水受益人口新增15万人。2015家农户草危房得到改造。农村扶贫8600户，脱贫5676户。实施农村最低生活保障制度取得新进展，近4万户困难农民家庭受益。

改革开放加快推进，沿江开发起步良好。市（区）属工贸企业“三置换一保障”改革基本完成，改制面达95%。乡镇企业改制结束。市属首批经营性服务类事业单位改革稳步推进。农村税费改革继续深化，农民负担监督工作得到加强。非公有制经济发展潜力进一步释放，新增私营企业3700多家，新增个体工商户近2万户，私营个体经济新增注册资本43亿元，完成税收11.2亿元。开放型经济发展势头良好。预计完成出口总额6.1亿美元，增长45.2%，增幅居于全省前列。在香港、厦门、上海等地成功举办了招商活动，全年合同利用外资4.8亿美元，实际利用外资3亿美元，同比分别增长53.6%和67.6%。泰兴新浦化工增资扩产项目、泰州开发区环保热电项目、韩国LG冰箱合资转独资等重大项目顺利落户。对外经济技术合作进一步扩大，预计新签对外承包劳务额1.8亿美元，完成营业额1.4亿美元，分别增长11.6%和18.4%。泰州边防检查站获准建立，海关、国检、海事等口岸查验机构为开放型经济发展作出了新的贡献。对外友好交往进一步扩大。沿江开发启动迅速，总体规划编制完毕，泰州港集装箱码头建成营运，沿江高等级公路、泰州电厂等重点基础设施项目进展顺利，杨湾海螺水泥等重点产业项目竣工投产。沿江开发园区建设力度加大，中石化（泰州）开发园区在泰州经济开发区正式挂牌，中国精细化工（泰兴）开发园区发展势头良好，高港科技工业园区初具规模，靖江市与江阴市两岸联动开发取得初步成效。

固定资产投资快速增长，城乡基础设施不断完善。预计完成固定资产投资230亿元，增长30%，增幅创近5年来新高。围绕“建设大城市，塑造新形象”目标，修编了中心城市总体规划。市第一污水处理厂一期、东风路二期等工程顺利推进，中心城市建设投资突破30亿元。市区新增绿地面积102万平方米，各类住宅竣工面积54.3万平方米。老城区一批后街背巷得到改造，7000多户居民受益。城建投资集团（有限）公司挂牌运行。城市建设项目决策、融资、实施、偿债、监督机制初步建立，城市经营水平有所提高。城市管理进一步加强。各市城区和重点中心镇建设步伐加快，实际完成投资43亿元，一批重点基础设施和综合开发项目顺利实施，城镇面貌有了较大改观。农田水利基础设施建设、沿江闸站改造、泰东河工程及城市防洪工程进展顺利。江平公路靖江段、泰常公路泰兴段、姜八公路姜堰段等干线公路改造、建设工程竣工通车，宁启铁路泰州段建设达到预定进度。新建、改造农村公路555公里。220千伏双墩（北郊）变电所和4座110千伏变电所建成投运。邮政、通信等基础设施建设也取得了新的进展。

社会事业协调发展，精神文明建设取得新成绩。成功举办了第四届科技经贸洽谈会。各级各类科技计划得到有效实施，企业技术创新体系建设继续加强，高新技术产业产值居全省第5位，科技进步对工业、农业增长的贡献率分别达41.7%和43.4%。人才高地建设得到加强，市留学人员创业园开始营运。教育改革进一步深化，素质教育水平进一步提高，高中阶段教育事业规模进一步扩大。市政府与省教育厅、南理工、南师大在泰州合作建立本科学院的协议已经签署。文化艺术创作取得新成果，我市获得省“五个一工程”组织工作奖及6项作品奖。淮剧《祥林嫂》入选上海国际艺术节，并晋京演出。在兴化成功举办中央电视台“心连心”艺术团慰问演出活动。重点文物修缮工程按计划实施，博物馆新馆启动建设。《泰州年鉴》（2003卷）编辑出版。对外宣传继续加强，新闻事业健康发展，《泰州晚报》创刊，各新闻单位为促进经济社会发展发挥了良好的宣传和导向作用。江苏省社科院泰州分院成立，并取得一批研究成果。去年4月以来，面对突如其来的“非典”疫情，全市上下万众一心，众志成城，构建了坚强有力的防控体系，夺得了“非典”防治工作的阶段性重大胜利。城乡公共卫生体系建设力度加大，市疾病预防控制中心业务楼建成。市中医院创建成三级中医院。农村卫生院改革与发展步伐加快。新型合作医疗制度农村居民覆盖面达68.8%。爱国卫生运动深入开展，泰兴市建成灭蝇先进城市，张郭镇通过国家卫生镇评审。质监、药监工作进一步加强，食品、药品放心工程有效推进。市体育馆主体工程封顶。计划生育市县乡服务所（站）全部建成，综合改革初显成效，人口出生率继续稳定在较低水平。土地市场秩序整顿和城市土地经营取得新成效，环境保护得到加强。外事、侨务工作为地方经济建设服务取得新成绩。民政、对台、旅游、统计、防震、气象、史志档案、民族宗教、老龄、妇女儿童、未成年人保护、残疾人等工作有了新进展。市救助管理站成立。群众性精神文明创建活动蓬勃开展，以创建全国文明城市工作先进市、国家卫生城市、国家环保模范城、全国双拥模范城和省级园林城市为主要内容的“五城同创”工作全面展开，并已进入全国双拥模范城行列。组织开展“沿江开发与泰州的跨越式发展”大讨论，促进了思想解放，激发了广大干部群众开发沿江、建设新泰州的热情。进一步加强国防教育，组织各市（区）国防动员委员会战时动员指挥演练和预备役团实兵实弹演习，圆满完成兵员征集任务。人民防空事业有了新发展。

民主法制建设继续推进，政府自身建设进一步加强。自觉接受市人大及其常委会的法律监督、工作监督和市政协的民主监督，重视和发挥各民主党派、工商联和无党派人士的参政议政作用。开展市人大代表建议、政协委员提案现场答复活动，建议、提案办理满意和基本满意率达99.1%。加强社区基层民主建设，建成43个示范社区。坚持依法行政，强化行政执法和执法监督，政府法制建设得到加强。普法教育继续开展，顺利通过全省“四五”普法中期检查。新型人民调解网络初步建立，信访工作进一步加强，化解了大量社会矛盾。积极推进“平安泰州”建设，“严打”斗争取得新成效，全市社会治安保持稳定。靖江市、高港区被评为省级安全市（区）。扎实抓好政府自身建设。全面开展学习型机关创建活动，努力提高政府机关工作人员素质。认真为民办实事，年初承诺为全市人民办的20件实事和为市区人民办的10件实事基本完成，年中启动的农村“5+1”实事工程（农村改水、草危房改造、建立新型合作医疗制度、新一轮农村公路建设、深化农村税费改革和农村低保）进展顺利，“南户北村”帮扶和机关干部结对帮扶城市贫困户活动取得成效。政务公开、行政监察、审计监督得到强化。对自来水价格和管道燃气价格调整等部分决策，实行了市民听证制度。全面推行行政事业性收费、涉农收费和涉及人民生活的收费公示制。开展公开议政活动，邀请失业人员再就业代表、群团组织和基层组织代表参加市政府常务会议，共商再就业工作大计。部门预算全面推行，政府采购制度不断完善，市本级国库管理制度改革试点进展顺利。机关后勤管理和社会化服务水平有了新提高。切实加强经济发展软环境建设，大力开展服务沿江开发主题监察活动，在40个部门和单位开展民主评议行风活动，并开设“行风热线”广播直播节目。市行政服务中心建成运行。加强廉政建设，党风廉政建设责任制进一步落实，领导干部廉洁自律工作继续强化，查处违法违纪案件工作取得新的进展，纠正医药购销、征地拆迁中的不正之风和教育乱收费等专项治理收到实效。

各位代表，过去一年取得的成绩，是在中共泰州市委的正确领导下，在市人大、市政协的监督和支持下，全市人民共同努力、艰苦奋斗的结果。在此，我谨代表市人民政府，向全市各行各业的广大干部群众，向各民主党派、工商联和无党派爱国人士，向中央和省驻泰机构以及人民解放军驻泰部队指战员、武警官兵和公安干警，向所有关心和支持泰州现代化建设的海内外朋友们表示衷心的感谢！

过去一年，全市经济和社会发展中还存在不少问题。主要是：农民收入增长缓慢，增收渠道尚需进一步拓宽；工业重大技改项目不多；服务业对经济增长的贡献份额偏低；实际利用外资总量仍然偏小；工业园区建设有待加快；中心城市的辐射和带动力较弱；城镇就业和再就业形势仍较严峻；精神文明建设、民主法制建设和政府自身建设还存在一些薄弱环节等等。对此，我们将高度重视，并采取有效措施，切实加以解决。

二、2004年主要任务

各位代表，今年是全面贯彻落实党的十六届三中全会精神，夯实力争“五年总量翻番、八年全面小康”基础的关键之年，经济社会发展面临着较为有利的条件和机遇。从国际环境看，全球经济逐步回升和结构调整加快，国际资本和产业将继续向中国转移；从国内形势看，我国正处于新一轮经济增长周期的扩张阶段，“长三角”地区成为国际资本和产业的集聚之地；从我市情况看，近年来经济快速增长的发展态势，企业改制的基本到位，沿江开发的不断加快以及一批重大项目的规划建设，为跨越发展提供了良好基础和有力支撑。更为重要的是，市委二届五次全体（扩大）会议提出的力争“五年总量翻番、八年全面小康”的奋斗目标和实施“六个倍增计划”(国内生产总值、全部工业增加值、有效投入、财政收入、外贸和自营出口、城乡居民收入倍增计划)的工作要求，已经并将进一步激发全市人民投身改革开放和现代化建设的巨大热情。同时，我们也应看到，今年电力、煤炭等部分生产要素供需矛盾日益突出，国家对出口退税、信贷和土地使用等宏观政策的适度调整可能对部分行业、领域产生影响。只要我们审时度势，真抓实干，在抢抓机遇中增创优势，在应对挑战中争取主动，在超越自我中寻求突破，就一定能够开创全市改革和发展新局面。

今年政府工作的总体要求是：以邓小平理论和“三个代表”重要思想为指导，全面贯彻党的十六大、十六届三中全会精神，认真落实市委二届六次全体会议的决策部署，牢固树立全面、协调、可持续的发展观，紧紧围绕力争“五年总量翻番、八年全面小康”的奋斗目标，以对外开放为第一动力，以沿江开发为突破口，以工业化为第一方略，加大改革力度，加快发展步伐，加速城市化进程，加强三个文明建设，促进经济持续快速协调健康发展和社会全面进步。

今年全市国民经济和社会发展主要调控目标初步安排为：国内生产总值增长12%以上，其中第一产业、第二产业、服务业增加值分别增长4%、15%和11%以上；财政收入增长18%；全社会固定资产投资增长20%；社会消费品零售总额增长11.5%；自营出口增长15%；实际利用外资4亿美元；城镇居民人均可支配收入增长9%，农民人均纯收入增长7%；城镇登记失业率控制在4.5%以内；人口自然增长率控制在4‰以内。

1、强力推进招商引资，促进开放型经济新跨越。把开放型经济作为推动全市经济跨越式发展的第一动力，落实关键措施，组织重点突破，努力实现率先跨越发展。

以扩大总量为主攻方向，推动引资大突破。围绕做大规模，坚持外资、内资齐招：紧紧抓住新一轮全球生产要素优化重组和产业转移的重大机遇，突出利用台资、注重日韩投资、扩大欧美投资，力求在招引境外资本上求突破；牢牢抓住“长三角”地区互动发展带来的重大机遇，主动承接上海、浙江和苏南等发达地区经济辐射，力求在招引南方资本上求突破。围绕提高招商成效，坚持“三个注重”：注重特色招商，以优势产业、优质资源招引外来资本；注重代理招商，抓紧建立和完善代理招商网络；注重以商引商，为现有外来投资企业和签约、开工项目提供高水准的服务。围绕提高招商组织程度，坚持“四个强化”：强化招商引资工作责任制，推动各级行政主要领导参与招商实践；强化招商队伍建设，提高招商人员综合素质；强化招商资源整合，全面推进农业、工业、服务业和社会事业、基础设施领域的对外招商；强化重大项目招商，建立快速反应、综合协调和跟踪服务机制。

以促进资本和产业集聚为目标，推动园区大发展。把加强园区建设作为今年开放型经济工作的重中之重，加快5个省级开发区和海陵、高港园区发展，推动春兰、扬子江等重点企业园区建设上水平，支持台商工业园、韩国工业园、俄罗斯工业园等主题园区发展，培育30个重点乡镇工业园区。统筹园区建设规划，努力彰显个性，错位发展；树立“大投入大产出”观念，改善园区基础设施和产业配套环境；引入“经营园区”理念，推进园区产业化经营、市场化运作；引导园区集中开发，集约发展，提高单位面积投资强度和综合效益；认真落实支持园区发展的各项政策措施，深入开展“五比五赛”活动，促进各类园区加快发展。

以市场开拓为龙头，推动外经贸大提高。积极应对国家外贸政策调整新形势，继续实施市场多元化、以质取胜和科技兴贸战略，千方百计扩大外贸出口。进一步调整出口产品结构，加大市场开拓力度；支持和指导更多企业取得和用好自营进出口权，鼓励企业采用国际标准组织生产，加强安全和环保认证工作；大力发展加工贸易和服务贸易，推进外贸方式多样化。继续实施“走出去”战略，鼓励具有比较优势的企业开展境外投资，推动外经企业扩大对外工程承包和劳务合作规模。抓紧建设泰州边检站。重视外事、侨务和对台工作，力争在对外交流合作上取得新进展。

2、突出抓好沿江开发，构建区域协调发展新格局。落实“统筹区域发展”要求，以沿江开发为突破口，促进沿江地区率先崛起，带动腹部地区开放开发，推动经济薄弱地区加快发展。

抢抓战略机遇，培育沿江经济增长极。加快沿江开发是江苏实现“两个率先”的重大战略举措，也是我市谋求跨越发展的重大机遇。围绕通过5-8年努力，把整个沿江地区建设成为承接国际制造业转移的新兴成长区，融入苏南、接轨上海、走向世界的先导区，南北联动、创新开发的示范区，全市跨越发展、率先崛起的支撑区的奋斗目标，将今年作为沿江开发的大推进之年，扣紧产业、园区、基础设施三个核心环节，突出抓好一批重点项目，力求沿江开放、开发取得重大突破。加快沿江化工、医药、机电、船舶修造、新材料等五大特色产业发展，培育沿江基础产业带；加快江阴开发区靖江园区建设和临江重点园区发展，狠抓环境营造、项目开发和产业投入，积极筹划和推动靖江与张家港、泰兴与常州、高港与扬中的联动开发；加快沿江高等级公路泰州段、泰州港码头、宁启铁路、泰州电厂等重大工程建设，做好泰州过江通道前期工作，构建发达的沿江基础设施网络。

呼应沿江发展，促进腹部地区开发。引导腹地市（区）、乡镇确立大沿江意识，强化区域经济联系和合作，在融入大沿江、共打沿江牌上做文章；策应沿江产业发展，主动做好产业配套，在拉长沿江产业链条上做文章；坚持基础设施“一盘棋”理念，强化与沿江交通等基础设施的对接和呼应，在改善和优化区域发展硬环境上做文章；找准承接辐射与激发内力的结合点，不断增强本地产业承接力和核心竞争力，在提升策应沿江开发能力上做文章。

实施“221工程”，加快黄桥老区和里下河地区经济薄弱乡镇发展。围绕到2007年，30个经济薄弱乡镇力争实现乡镇财政和村级经济收支“两个基本平衡”、人均国内生产总值和人均财政收入“两个翻番”、农民人均纯收入年增幅高于全市平均水平的目标，坚持上下联动、领导挂帅、部门挂钩和结对帮扶，落实工作目标和相关责任，落实扶持政策和扶持资金；坚持优先支持经济薄弱乡镇基础设施和社会事业建设，进一步改善发展环境；坚持启动内因、增强内力，引导经济薄弱乡镇把发展私营个体经济、促进农村劳动力输出、培育地区特色产业等关键措施落实到位，努力实现30个经济薄弱乡镇国内生产总值、预算内财政收入和农民人均纯收入增幅高于全市平均水平。

3、进一步加快改革步伐，增创经济体制新优势。认真贯彻党的十六届三中全会精神，落实“五个统筹”，恪守“五个坚持”，把经济体制改革进一步引向深入，为跨越发展提供体制保障。

毫不动摇地发展非公有制经济。紧紧抓住党的十六届三中全会给非公有制经济发展带来的重大机遇，认真贯彻省委优先发展非公有制经济的重要决策，在继续积极吸引外来民间资本的同时，努力激活和壮大本市非公经济的内在力量。大力培育和弘扬创业精神，鼓励本地在外能人回乡创业，引导广大群众投资兴业；认真落实“六放”的要求，营造宽松的创业环境；在推动非公有制经济“铺天盖地”发展的同时，引导和支持其加快体制创新、科技创新和管理创新，尽快培育一批“顶天立地”的私营企业集团，力争今年销售收入1000万元以上的私营企业达到400家，其中亿元以上的15家；推动私营企业向园区集中，培育企业集群和特色产业板块；完善政策扶持、信用担保、信息咨询、权益保护体系，为民间资本创业和非公经济发展提供优质高效服务。

继续深化企业改革。按照“突出重点、猛攻难点、整体推进”的思路，对尚未改制的重点企业和困难企业，强化组织领导，强化协调服务，确保年内全面完成市（区）属工贸企业“三置换一保障”改革任务；按照建立“归属清晰、权责明确、保护严格、流转顺畅”的现代产权制度的方向，引导已改制企业促进股权流动和重组，建立有效制衡的法人治理结构，增强创新创业的动力；按照转换机制的要求，指导改制企业进一步深化内部劳动用工、人事和收入分配制度改革。基本完成市（区）属建筑、交通企业“三置换一保障”改革，加快推进其他行业企业产权制度改革。积极探索国有资产监管和经营的有效形式，促进保值增值。

加快推进事业单位改革。按照“保住一头，放开一片”的原则和“分类定位、分开管理、分别改革”的思路，抓好事业单位改革。对管理类事业单位，抓好清理、归并、精简和规范，逐步弱化和移交管理职能；对公益类事业单位，抓好布局调整和结构优化，深化内部三项制度改革；对经营性服务类事业单位，大力推进“三置换一保障”改革，力争年内基本完成改制任务。深化水利工程管理体制改革，完成经营性单位改制，推进公益性、准公益性单位改革。

加强劳动就业和社会保障工作。就业是民生之本，稳定之本。把扩大就业放在经济社会发展更加突出的位置，实施积极的就业政策，努力改善创业和就业环境，力争全年城镇失业人员再就业2.2万人以上。注重发展劳动密集型产业，注重扶持中小企业，注重发展非公有制经济，注重采用灵活多样的就业形式，千方百计增加就业岗位，拓宽就业渠道；加大再就业资金筹措力度，继续推进政府差额购买就业岗位和培训成果，帮助特殊困难群体就业再就业；完善就业服务体系，加强职业教育和技能培训；认真落实各项优惠政策和措施，鼓励各类企业吸纳失业人员再就业，鼓励失业人员自谋职业、自主创业。组织实施市区“金保”工程，年底实现养老保险系统与省实时联网；大力推进非公有制企业职工养老保险全覆盖，健全失业保险制度；合理控制参保人员个人负担，进一步扩大医疗保险覆盖面，力争退休人员全部进入医保；建立困难企业职工大病医疗保险，落实城乡居民最低生活保障制度。强化社会保障基金征缴和监管，确保基金安全。继续发展社会救灾、救济、互助、福利等社会保障事业。

4、着力推进产业发展，促进经济运行质量新提高。

实施倍增计划，推动工业提速增效。把工业化作为泰州富民强市的第一方略，突出抓好有效投入、高新技术产业发展、规模企业培育、企业信息化建设和品牌战略实施，加速新型工业化进程，全年列统工业增加值增长16%以上。把扩大有效投入作为支撑工业强势发展的重要抓手，通过优化项目服务、发展银企合作、落实技改贴息，支持140个投入3000万元以上重点技改项目的实施，确保全年技改投资增长30%；把发展高新技术产业作为促进新兴产业成长的重要举措，加快高新技术产品开发和高新技术企业发展，推动200个年新增销售3000万元以上重点新产品的开发；把培植规模企业作为提升工业整体水平的重要突破口，落实促进30强企业跨越发展激励措施，培育100家以上销售过亿元或利税过千万元企业，同时继续引导和支持中小企业向“专、精、特、新”方向发展；把推进企业信息化作为工业结构调整的重要着力点，在列统企业加快推行管理信息化，运用信息技术改造和提升传统产业；把实施品牌战略作为增强企业综合竞争力的重要途径，坚持质量兴市，鼓励企业争创名牌产品和驰名商标。

按照“三化一转移”（农村城镇化、农业产业化、农民知识化，转移农村劳动力）的思路，进一步拓宽农民增收渠道，加快农业和农村经济发展。把解决好“三农”问题作为全局工作的重中之重，把农民致富放到更加突出的位置。增加农业投入，加大“三资”开发农业力度，力争实际利用“三资”10亿元。加快农业园区发展，重点抓好市农业开发区产业发展研究、总体规划完善和基础设施建设，年内投入1亿元以上。扶持农业产业化龙头企业加快发展，对技改投入达到一定规模的项目给予贷款贴息。积极培育各类农产品市场竞争主体，带动农业产业化经营和农业结构调整。加快无公害农产品基地建设，建成市农产品质量检测中心。抓好动植物疫病防治，保障农产品生产安全。把扩大劳务输出作为现阶段最大的农民致富工程，进一步改善农村劳动力转移环境，建立健全农村劳动力培训机制，抓紧搭建各类劳务输出平台，强化外出劳务人员权益保障，全年新增劳务输出8万人。按照“依法、自愿、有偿”原则，流转土地承包经营权。改革土地征用制度，加快建立土地换社保的补偿机制，确保失地农民的长远生计。落实取消农业特产税政策，深化农村税费配套改革，切实控减农民负担。完成农业技术推广服务体系改革，促进农业可持续发展。

加快服务业发展，稳定提高其在国民经济中的比重。积极创新商贸流通经营业态，大力发展连锁经营、物流配送和专业大市场。活跃住房二级市场和租赁市场，推进住房货币化改革，规范和完善住房公积金制度，促进房地产业健康成长。按照产业化、连锁化、品牌化、标准化的方向，加快发展社区服务业。把旅游业作为新的增长点来培育，抓紧编制全市旅游总体规划，整合旅游资源，发展特色旅游。推进信息技术在各行各业的广泛运用，扩大信息消费。发展文化、体育产业，培育和规范会计、审计、律师、公证、评估、咨询等中介组织。用好引导资金，支持服务业重点项目建设。

着力培大育强，提升建筑业整体发展水平。围绕“经济指标翻一番，培植两个双百亿，队伍突破五十万，农民人均增千元”的五年目标，抓住北京办奥、上海办博等重大机遇，促进泰州由建筑大市向建筑强市转变，全年建筑业总产值增长15%以上。加快泰兴、姜堰两个省级“建筑之乡”发展，指导靖江、兴化和海陵、高港建筑业扩容升档，推进江苏一建、江苏中兴、江苏正太等龙头企业发展。继续引导企业创优升级，加大市场开拓力度，加快建筑技术进步，加强与国外大型建筑企业联合，在竞争与合作中加速发展。

做好财税金融工作，增强政府的调节和保障功能。坚持培植财源与经济发展并重、收入征管和支出控制并重、加大信贷投入与强化信用意识并重，发挥财税金融对经济发展的促进作用。重视财源建设，坚持依法治税，保证财政收入稳定增长。加强预算管理，坚持保运转、保稳定、保重点的原则，确保社会保障和教育、科技、农业等重点支出。全面推行国库集中收付制度。重视乡镇财政建设，完善管理体制，严格依法理财，保障基层政权建设，促进经济社会协调发展。调整优化信贷结构，强化对重大项目、非公有制经济和农业结构调整的支持。进一步加强审计监督。继续整顿和规范市场经济秩序，打造“诚信泰州”。做好统计工作，加强对经济运行的分析监测。

5、大力推进城乡建设、精神文明和民主法制建设，营造跨越发展新环境。适应改革开放新形势，统筹城乡发展，统筹经济社会发展，统筹人与自然和谐发展，协同推进三个文明建设。

大跨度推进中心城市建设，继续加大城乡基础设施投入。实施城市建设三年纲要，以更大手笔、更快节奏，掀起新一轮中心城市建设高潮。在城市规划上，注重增强前瞻性和开放性，抓好专业规划和详细规划的制定，充分发挥其指导和调控作用；在城市建设上，重点抓好市区东城河风景区、泰山公园改造、铁塔广场、稻河整治、城市防洪、污水与垃圾处理等总投资达150亿元的94项重点工程，当年完成投资49亿元，完善城市功能，提升城市品位；在城市经营上，完善决策、融资、偿债、实施、监督五大机制，推进城建投资集团规范、高效运作；在城市管理上，推行综合执法，下移管理重心，提高管理绩效。继续引导各市城区、重点中心镇抓好一批重点工程建设，加快发展步伐，完善市域城镇体系。加大城乡基础设施建设力度。完成姜八公路泰兴段、334省道泰兴绕城段、328国道姜堰绕城段、高兴东公路兴化城区过境段等改建、续建工程，建成农村公路1000公里。加快泰东河工程、里下河灾后重建等重点水利工程建设，抓好农村险桥改建、河道疏浚。完善电力、通信等基础设施，建成500千伏泰兴变--斗山变线路工程和220千伏、110千伏变电所各3座，实施电话交换机扩容、移动G网和联通C网扩容工程。

加强精神文明建设，繁荣各项社会事业。扎实推进“五城同创”，确保通过国家卫生城市省级调研，通过环保模范城市国家调研。加快沿江精神文明带建设，推动“文明跨江行动”深入开展。进一步抓好“双拥共建”活动，切实做好国防教育、民兵预备役和征兵工作。启动建设市级人防指挥所和国防教育园。大力实施“科技兴市”战略，继续推进人才、挂靠、创办、开发和科普工程，抓好国家级高新技术创业服务中心建设。创新人才工作机制，落实人才激励政策，培育、吸引和用好各类人才，构建人才高地。巩固和完善“以县为主”农村义务教育管理体制，继续提升普通高中教育水平，加强职业技术教育和培训，重视成人教育。完成市莲花学校新校区一期工程。大力发展高等教育，开工建设牧院等三所高校新校区，确保南理工泰州科技学院、南师大泰州文理学院年内招收本科学生。促进民办教育事业健康规范发展。完善资助家庭经济困难学生入学制度。加强农村、社区文化建设，繁荣和净化文化市场。完成市博物馆新馆主体工程。继续挖掘和整合历史文化资源，加大保护和开发力度。积极发展有线数字电视，提高农村有线广播电视入户率。建立“非典”长效防治工作机制，完善各类突发性公共卫生事件医疗救治网络。启动建设市传染病医院。规范社区卫生服务，加强城乡公共卫生体系建设，扩大农村新型合作医疗覆盖面和受益面。推进区域供水工程。实施全民健身计划纲要，办好市第二届运动会，建成市体育中心一期工程。重视民族宗教工作，实施光孝寺扩建、城隍庙修缮工程。积极发展新闻、气象、抗震、科普、史志档案事业，做好妇女儿童、老龄和残疾人工作。认真贯彻落实计划生育、保护环境和保护资源基本国策，建立计划生育社会保障机制和人口问题综合治理机制，强化工业污染防治、环境综合整治和生态环境建设，实行严格的耕地保护制度，促进可持续发展。

继续推进民主法制建设，努力维护社会稳定。自觉接受同级人民代表大会及其常委会的监督，支持人民政协履行政治协商、民主监督、参政议政职能，加强与各民主党派、工商联、无党派人士联系，充分发挥工会、共青团、妇联等人民团体的桥梁纽带作用。认真办理人大代表建议和政协委员提案。进一步健全基层民主制度，加强社区建设，抓好全市第7届村委会换届选举工作。坚持依法治市，推进“法治泰州”建设。扎实抓好“四五”普法，强化法律援助，加强人民调解工作，不断完善法律服务体系。全面推进“平安泰州”建设，落实社会治安综合治理措施，依法打击各种违法犯罪，切实加强社会治安防控体系建设，防范和惩治“法轮功”等邪教组织，坚决扫除社会丑恶现象。认真落实安全生产责任制，严防重、特大事故发生。

６、切实加强自身改革与建设，开创政府工作新局面。按照“为民、务实、清廉”要求，加强政府自身改革与建设，不断提高统揽全局、统筹发展的能力和公共管理、公共服务的能力，更好地肩负起改革发展稳定的重任。

坚持与时俱进，努力建设学习型政府。把加强学习作为一项紧迫任务，全面开展学习型机关、学习型班子、学习型干部创建活动，逐步向学习型城市、学习型社会迈进。引导和组织全体公务员加强学习，做到学以立德、学以增智、学以致用。深入学习邓小平理论、“三个代表”重要思想和十六大、十六届三中全会精神，着力在掌握理论精髓上下功夫，加强对新情况、新问题、新矛盾的研究，提高观察问题、分析问题、解决问题的能力；深入学习市场经济、现代科技、法律法规等方面的知识，着力在学深悟透上下功夫，提高运用改革办法、市场手段推进工作的能力；深入学习先进地区的创新思维、基层群众的大胆创造，着力在创造性运用上下功夫，努力提高与时俱进、开拓创新的能力。

坚持执政为民，努力建设服务型政府。把为民、利民、便民作为全部工作的立足点，努力把代表最广大人民群众根本利益的要求体现在行动上，落实到服务中。深入基层、深入一线，多谋富民之策、多办利民实事，确保农村“5+1”实事达到预定进度，坚决完成向人民承诺的10件实事。带着感情，带着责任，倾听百姓疾苦，化解百姓困难，尤其是对社会弱势群体给予更多关心和扶持。认真落实信访工作领导责任制和领导干部接待群众来访制，妥善处理人民内部矛盾特别是涉及群众切身利益的矛盾。加快政府职能转变，切实将主要精力放到为市场主体服务和创造良好发展环境上来。进一步加强经济发展软环境建设，以企业和群众呼声为第一信号，着力从反映强烈的问题入手，集中整治，建章立制，努力让群众满意、基层满意和投资者满意。继续改善和优化对外来投资者服务，办好外商会馆。坚持依法行政，严格按照法定权限和程序履行职责、行使权力、接受监督，增强行政行为的公信力。以实施《行政许可法》为契机，深化行政审批制度改革，依法设定行政许可项目，推行集中办理和联合办理。抓好市行政服务中心规范、高效运作。加快电子政务建设。继续扩大政务公开，完善政府重大经济社会问题的科学化、民主化、规范化决策程序，增加透明度和公众参与度。把评判政府服务质量和工作绩效的权力交给群众、交给企业、交给投资者，开展行风评议，办好行风热线。强化行政监察，改善行政管理，提高行政效率，确保政令畅通。

坚持求真务实，努力建设实干型政府。把实干作为一种工作作风、一种思想境界、一种政治责任来认识和对待，引导政府组成人员和全体工作人员锁定“411工程”（全市经济社会发展40项目标，城建10项重点工程和为人民办10件实事），沉下心来、扑下身子，落实措施、落实责任，抓紧每一天，走好每一步，脚踏实地不浮躁，埋头苦干不张扬。结合工作实际，切实将市委决策部署细化为可操作的办法和措施，一件一件地落实，一项一项地完成。惜时如金，以强烈的紧迫感，雷厉风行地抓落实。居安思危，勇于面对困难，善于化解矛盾，着力在推进重点工作上求实效，在化解难点问题上求突破。树立正确的政绩观，坚决不搞急功近利的“形象工程”和劳民伤财的“政绩工程”，力戒形式主义和官僚主义。建立健全重实干、重实绩的考评机制，使勤政为民、求真务实的干部得到褒奖，使好大喜功、弄虚作假的干部受到惩戒，在政府机关形成勤政为民、踏实苦干的浓厚风气。

坚持从严治政，努力建设廉洁型政府。认真落实廉政建设各项规定，深入开展反腐败斗争。教育政府机关全体公务员树立正确的世界观、人生观、价值观，时刻牢记“两个务必”要求，做到不为名利所困，不为物欲所诱，不为人情所扰，经得起各种考验。加强制度建设，努力从源头上预防和治理腐败。完善监督机制，做到权力运行到哪里，监督就延伸到哪里，形成贯穿权力实施全过程的监督体系。严格落实党风廉政建设责任制，一级抓一级、一级带一级，逐级负责，层层落实。深入开展纠风和专项治理工作，着力解决群众反映强烈的突出问题，树立清正廉洁的良好形象。

各位代表，面对新形势、新机遇、新任务，我们深感责任重大。让我们紧密团结在以胡锦涛同志为总书记的党中央周围，高举邓小平理论伟大旗帜，自觉实践“三个代表”重要思想，在中共泰州市委的领导下，紧紧依靠全市人民，与时俱进，开拓创新，团结拼搏，扎实工作，为完成全年各项目标任务，推进“五年总量翻番、八年全面小康”进程而努力奋斗！
